# Impaction of an Endobronchial Watanabe Spigot Within a Tracheostomy Tube

**DOI:** 10.1002/rcr2.70567

**Published:** 2026-03-26

**Authors:** Takumi Fukaya, Taro Koba, Miake Yamamoto, Mikio Takamori

**Affiliations:** ^1^ Department of Respiratory Medicine and Oncology Tokyo Metropolitan Tama Medical Center Tokyo Japan

**Keywords:** airway obstruction, bronchial occlusion, endobronchial Watanabe spigot, spigot migration, tracheostomy tube

## Abstract

A 78‐year‐old woman underwent endobronchial Watanabe spigot (EWS) placement for post‐cryobiopsy bleeding and subsequently required a tracheostomy. Two months later, a migrated EWS was incidentally found impacted within her uncuffed tracheostomy tube. This case highlights the importance of follow‐up imaging and careful inspection of tracheostomy tubes for possible spigot impaction.

## Graphical abstract

We describe a case of endobronchial Watanabe spigot (EWS) impaction within a tracheostomy tube, a complication not previously reported. This case highlights the importance of follow‐up imaging and careful inspection of tracheostomy tubes for possible spigot impaction.

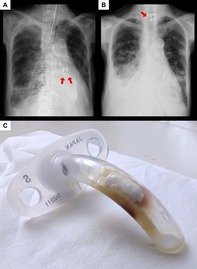

1

A 78‐year‐old woman presented with multiple pulmonary nodules, mediastinal lymphadenopathy and right pleural effusion. Transbronchial cryobiopsy of the left lower lobe resulted in massive bleeding requiring emergency intubation, veno‐venous extracorporeal membrane oxygenation (ECMO) and bronchial artery embolisation. Endobronchial Watanabe spigots (EWSs) were subsequently placed in the left lateral basal segmental bronchus (B9) and posterior basal segmental bronchus (B10) to prevent rebleeding (Figure 1A).

**FIGURE 1:**
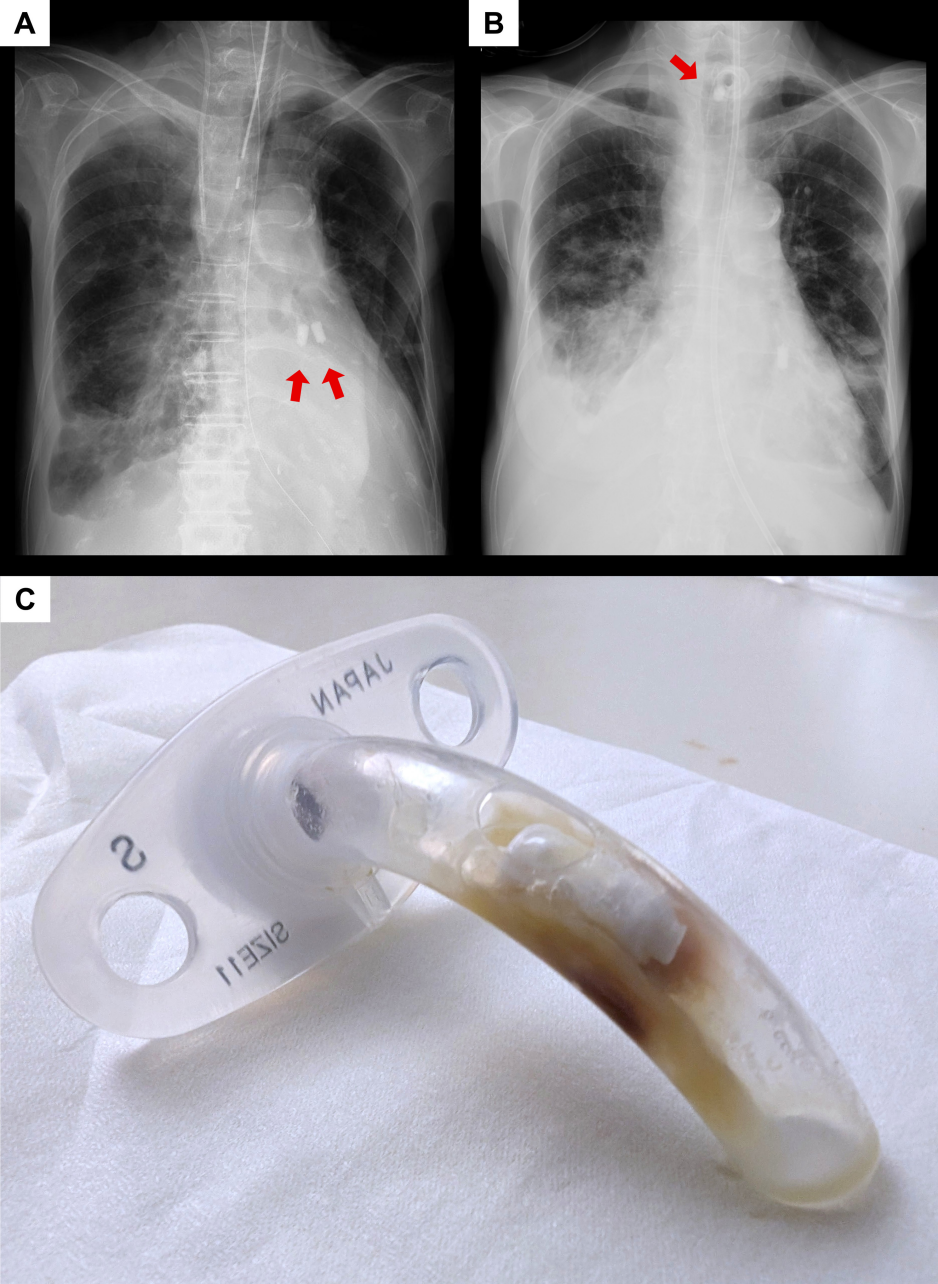
(A) Portable chest radiography (anteroposterior view, supine) demonstrated the initial placement of endobronchial Watanabe spigots in the left lateral basal segmental bronchus (B9) and posterior basal segmental bronchus (B10) (arrows). (B) Chest radiography (posteroanterior view, upright) demonstrated a foreign body (arrow) within the tracheostomy tube. (C) The migrated endobronchial Watanabe spigot (EWS) can be seen obstructing the lumen of the removed tracheostomy tube.

After successful weaning from ECMO, a tracheostomy was performed. Two months later, routine chest radiography revealed a foreign body within the uncuffed single‐lumen tracheostomy tube (Figure 1B) despite the absence of respiratory symptoms. A migrated EWS was found impacted within the removed tube (Figure 1C).

Spigot migration is a known complication of endoscopic bronchial occlusion using an EWS [1]; however, the impaction of a spigot within a tracheostomy tube has not previously been reported. In the present case, the tracheostomy tube lumen was nearly completely obstructed by the migrated EWS and adherent sputum. Nevertheless, because the tube was uncuffed, airflow through the upper airway was preserved, thus preventing acute airway obstruction. Had a cuffed tracheostomy tube been in place, fatal suffocation might have occurred. Clinicians should consider follow‐up chest imaging after EWS placement, particularly in tracheostomised patients, and carefully inspect not only the lung fields but also the tracheostomy tube for possible spigot impaction.

## Author Contributions

Takumi Fukaya contributed to the conception of the work, patient management, data acquisition and interpretation, drafting of the manuscript and final approval of the version to be published. Taro Koba, Miake Yamamoto and Mikio Takamori contributed to patient management, interpretation of data, critical revision of the manuscript for important intellectual content and final approval of the version to be published. All the authors agree to be accountable for all aspects of the work.

## Funding

The authors have nothing to report.

## Ethics Statement

Ethical approval was not required for this case report in accordance with institutional policy and national regulations.

## Consent

The authors declare that written informed consent was obtained for the publication of this manuscript and accompanying images using the consent form provided by the Journal.

## Conflicts of Interest

The authors declare no conflicts of interest.

## Data Availability

Data sharing is not applicable to this article as no datasets were generated or analysed during the current study.

## References

[1] D. Himeji , G. I. Tanaka , C. Fukuyama , R. Shiiba , A. Yamanaka , and K. Beppu , “Clinical Evaluation of Endoscopic Bronchial Occlusion With an Endobronchial Watanabe Spigot for the Management of Intractable Pneumothorax, Pyothorax With Bronchial Fistula, and Postoperative Air Leakage,” Internal Medicine 59, no. 15 (2020): 1835–1839. 32350193 10.2169/internalmedicine.3900-19 PMC7474981

